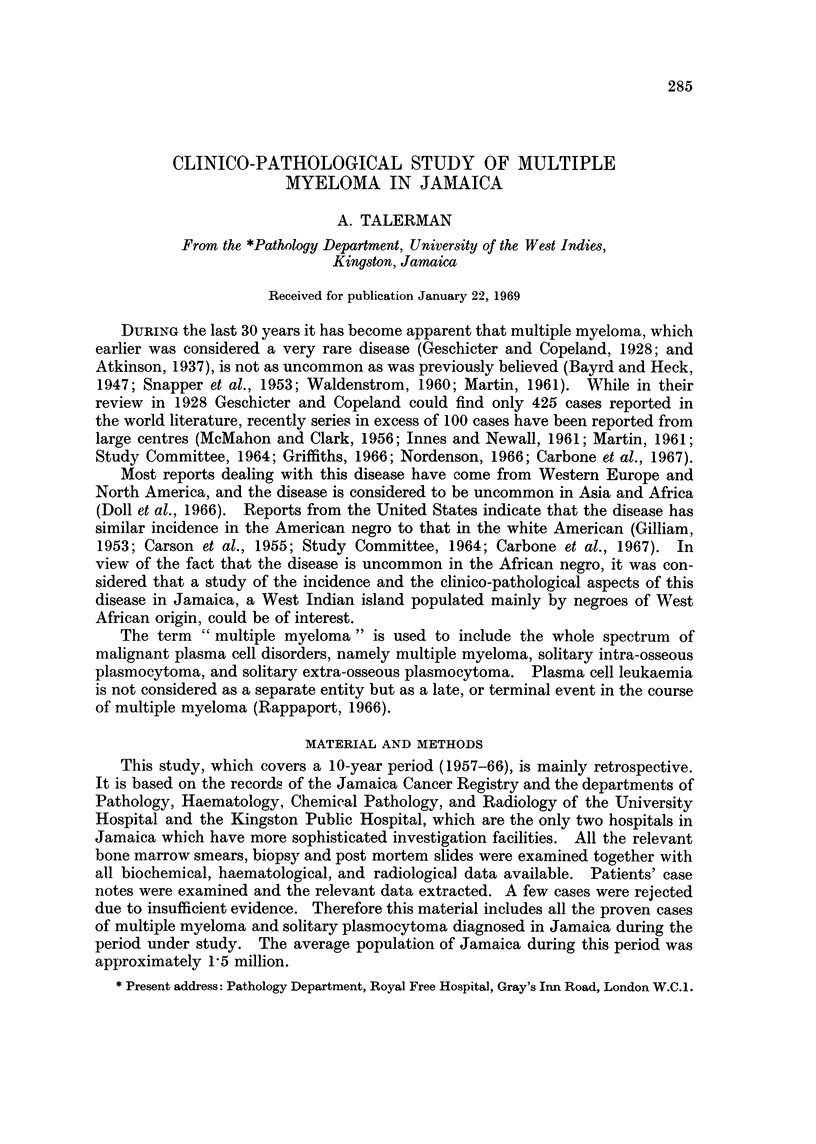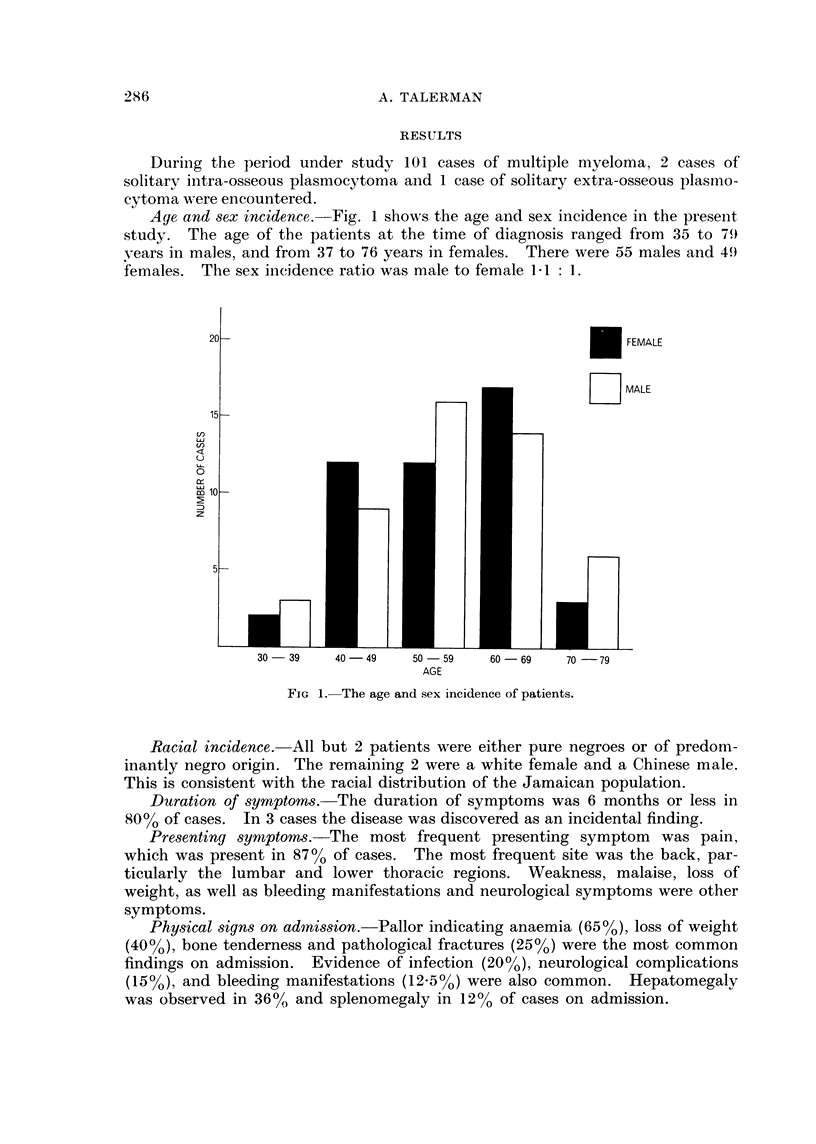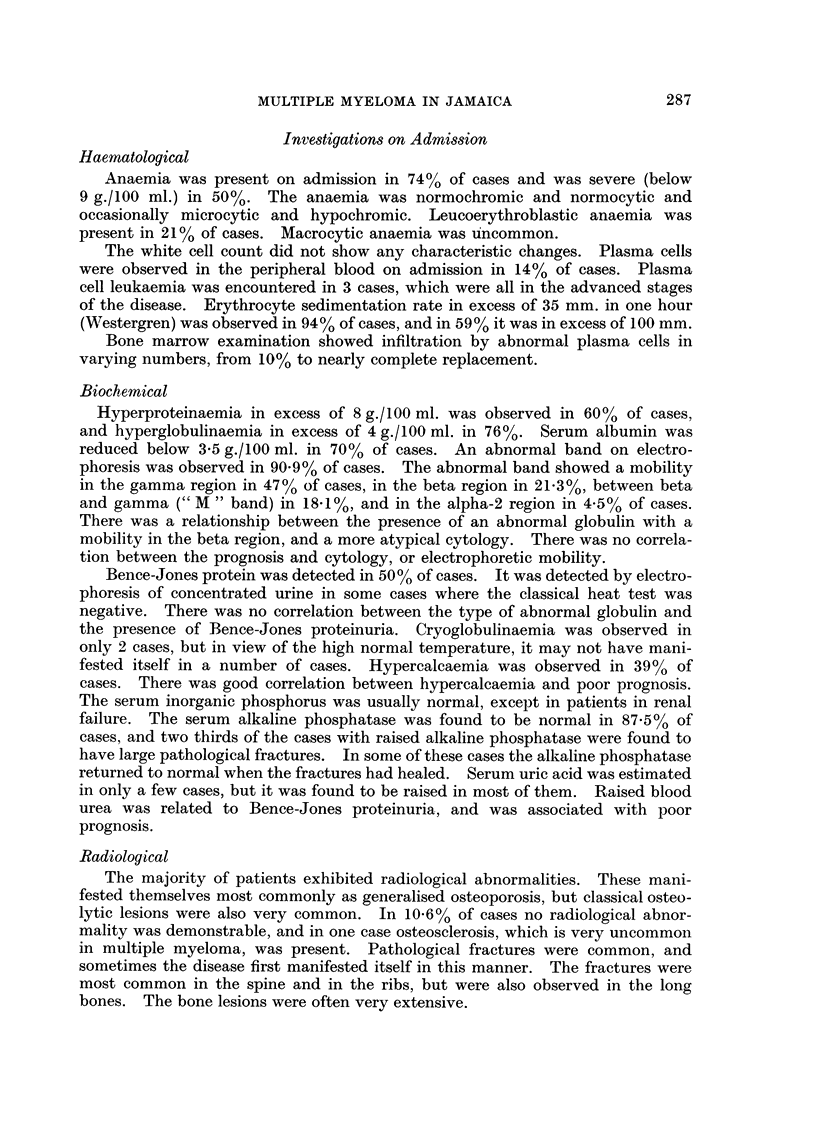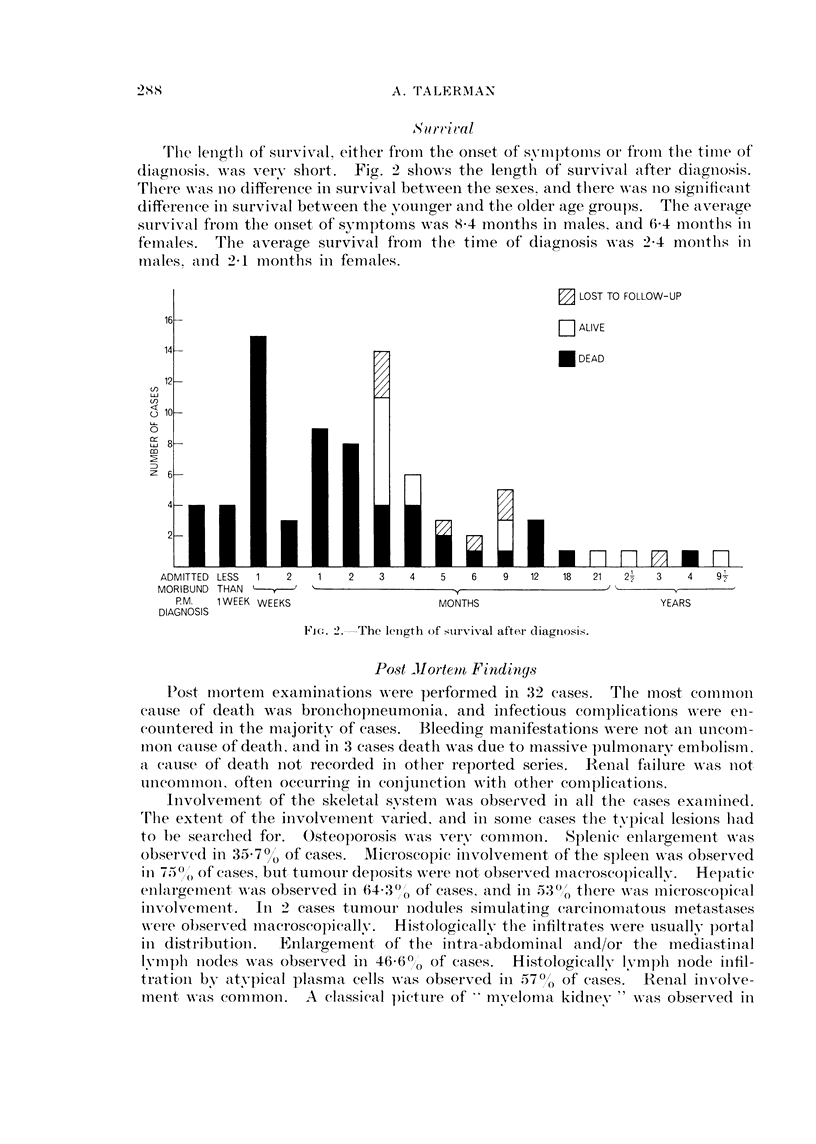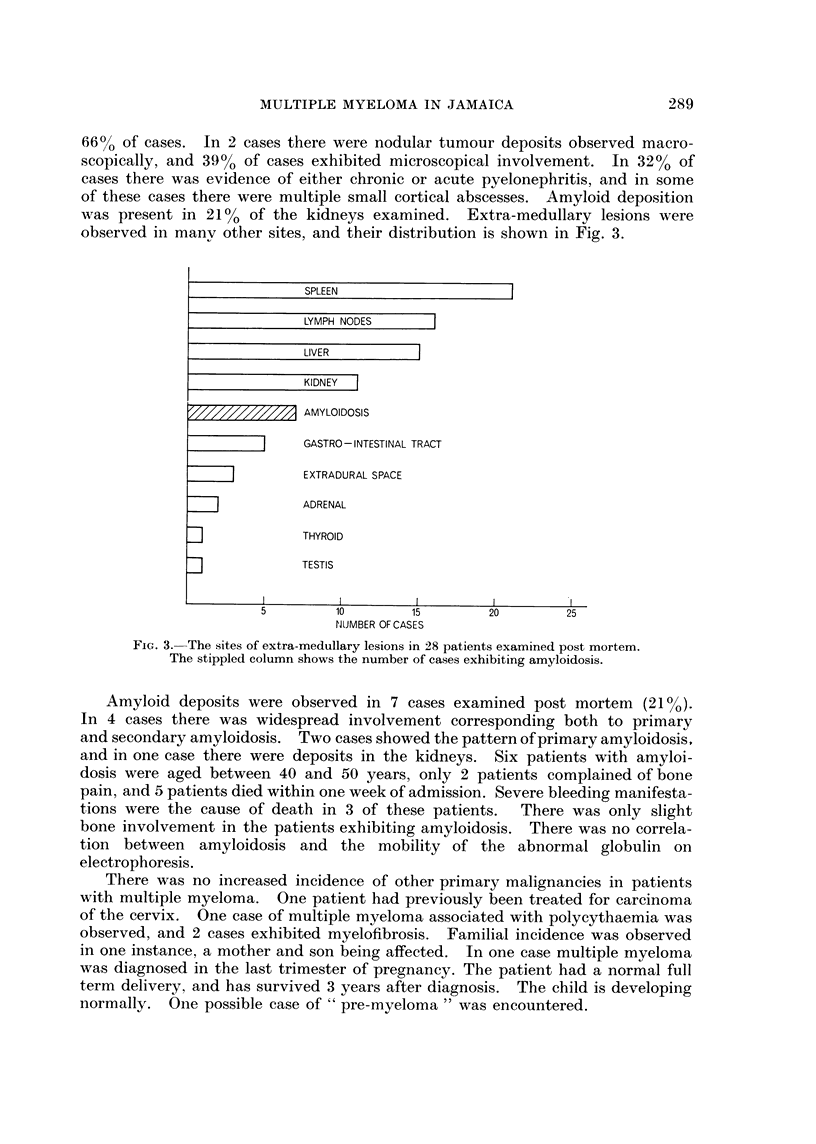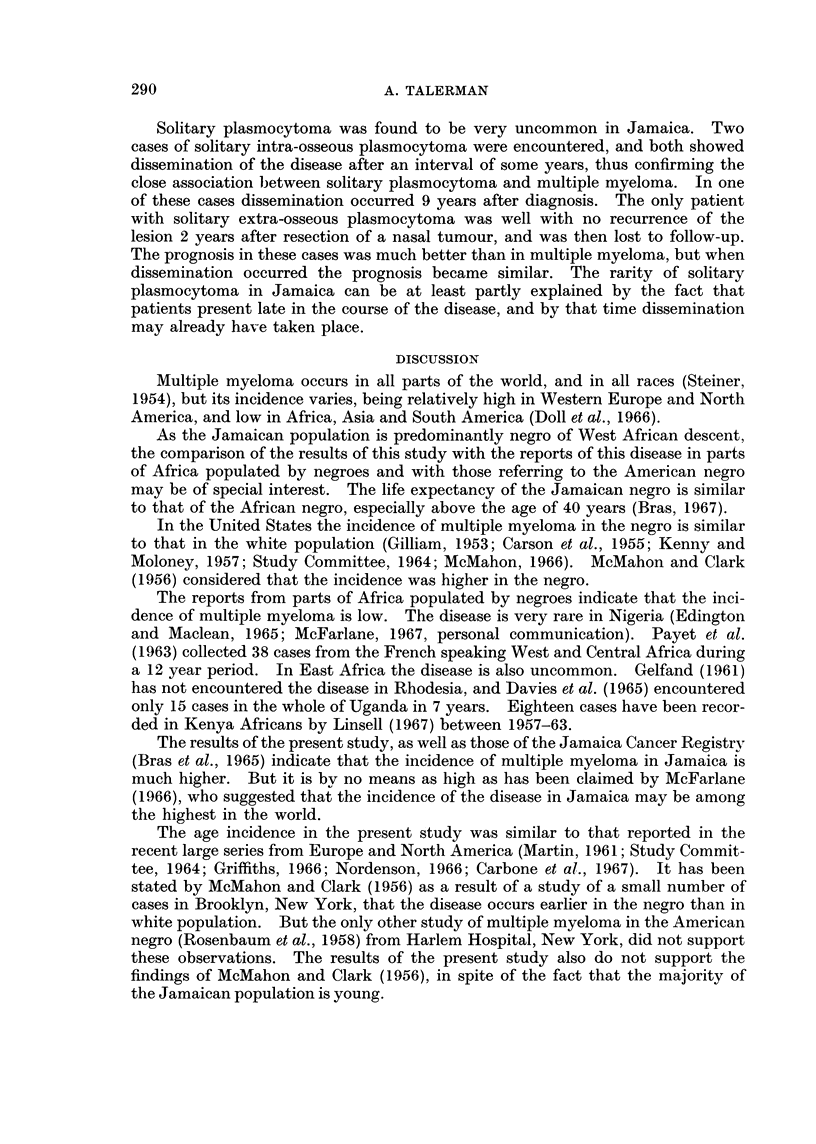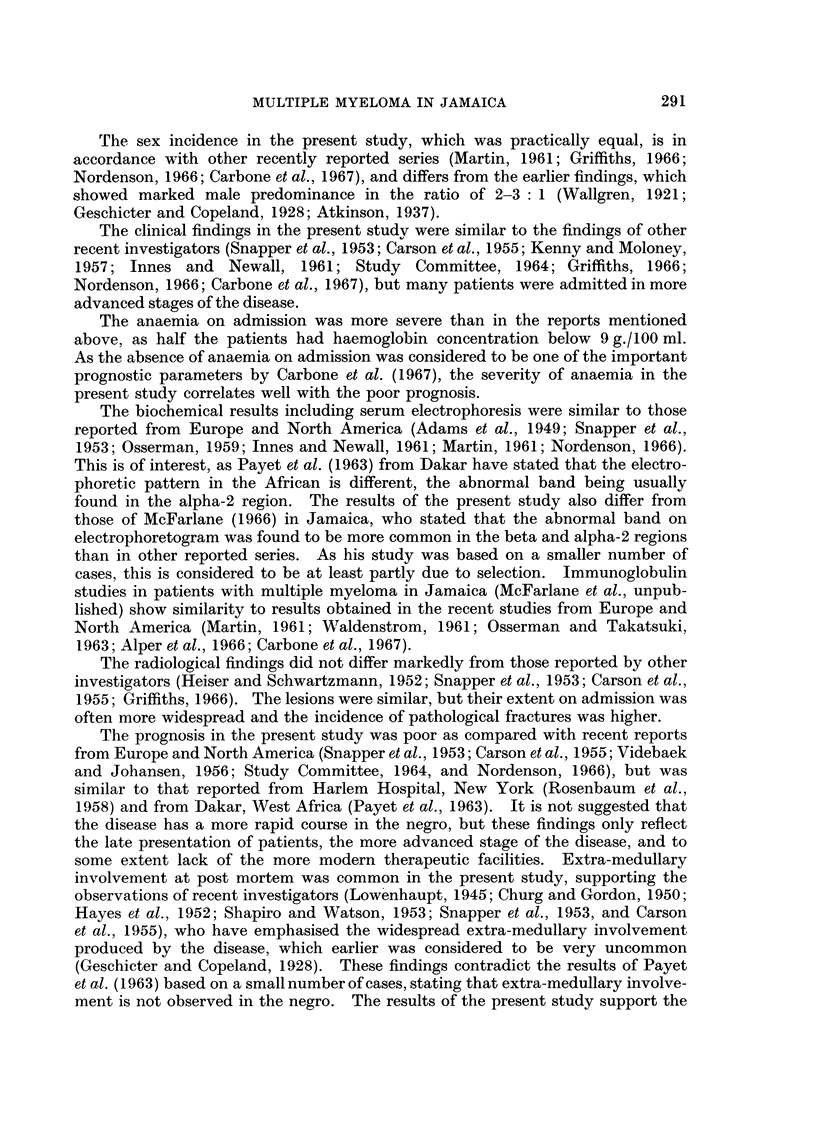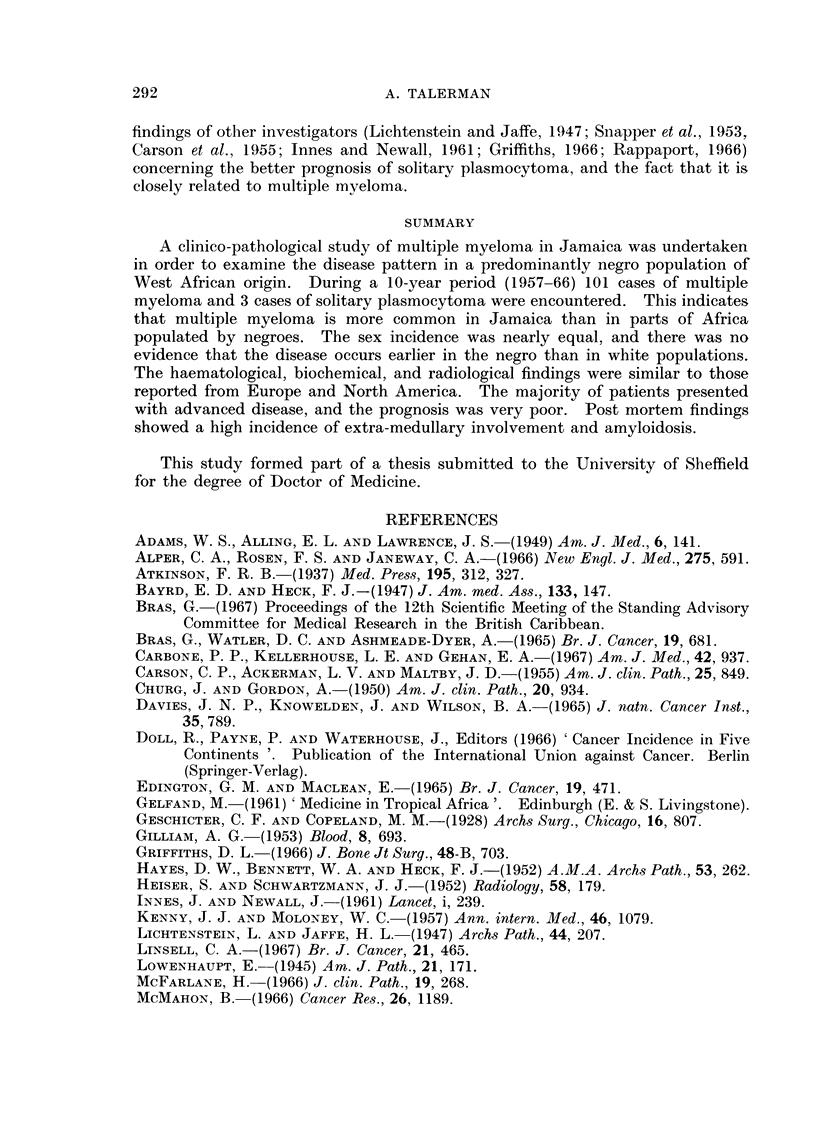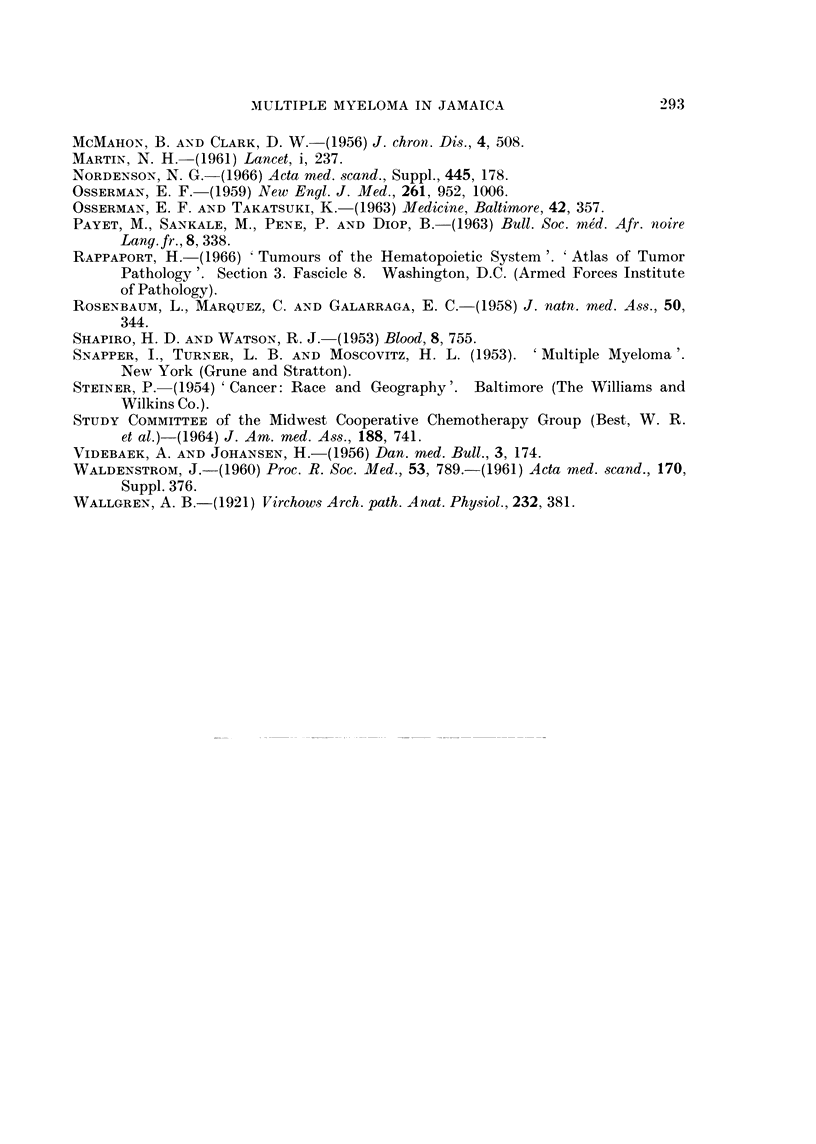# Clinico-pathological study of multiple myeloma in Jamaica.

**DOI:** 10.1038/bjc.1969.38

**Published:** 1969-06

**Authors:** A. Talerman


### CLINICO-PATHOLOGICAL STUDY OF MULTIPLE MYELOMA IN JAMAICA

A. TALERMAN

*From the *Pathology Department, University of the West Indies, Kingston, Jamaica*

Received for publication January 22, 1969

DURING the last 30 years it has become apparent that multiple myeloma, which earlier was considered a very rare disease (Geschicter and Copeland, 1928; and Atkinson, 1937), is not as uncommon as was previously believed (Bayrd and Heck, 1947; Snapper *et al.*, 1953; Waldenstrom, 1960; Martin, 1961). While in their review in 1928 Geschicter and Copeland could find only 425 cases reported in the world literature, recently series in excess of 100 cases have been reported from large centres (McMahon and Clark, 1956; Innes and Newall, 1961; Martin, 1961; Study Committee, 1964; Griffiths, 1966; Nordenson, 1966; Carbone *et al.*, 1967).

Most reports dealing with this disease have come from Western Europe and North America, and the disease is considered to be uncommon in Asia and Africa (Doll *et al.*, 1966). Reports from the United States indicate that the disease has similar incidence in the American negro to that in the white American (Gilliam, 1953; Carson *et al.*, 1955; Study Committee, 1964; Carbone *et al.*, 1967). In view of the fact that the disease is uncommon in the African negro, it was considered that a study of the incidence and the clinico-pathological aspects of this disease in Jamaica, a West Indian island populated mainly by negroes of West African origin, could be of interest.

The term " multiple myeloma " is used to include the whole spectrum of malignant plasma cell disorders, namely multiple myeloma, solitary intra-osseous plasmocytoma, and solitary extra-osseous plasmocytoma. Plasma cell leukaemia is not considered as a separate entity but as a late, or terminal event in the course of multiple myeloma (Rappaport, 1966).

### MATERIAL AND METHODS

This study, which covers a 10-year period (1957–66), is mainly retrospective. It is based on the records of the Jamaica Cancer Registry and the departments of Pathology, Haematology, Chemical Pathology, and Radiology of the University Hospital and the Kingston Public Hospital, which are the only two hospitals in Jamaica which have more sophisticated investigation facilities. All the relevant bone marrow smears, biopsy and post mortem slides were examined together with all biochemical, haematological, and radiological data available. Patients' case notes were examined and the relevant data extracted. A few cases were rejected due to insufficient evidence. Therefore this material includes all the proven cases of multiple myeloma and solitary plasmocytoma diagnosed in Jamaica during the period under study. The average population of Jamaica during this period was approximately 1·5 million.

* Present address: Pathology Department, Royal Free Hospital, Gray's Inn Road, London W.C.1.

### RESULTS

During the period under study 101 cases of multiple myeloma, 2 cases of solitary intra-osseous plasmocytoma and 1 case of solitary extra-osseous plasmocytoma were encountered.

*Age and sex incidence.*—Fig. 1 shows the age and sex incidence in the present study. The age of the patients at the time of diagnosis ranged from 35 to 79 years in males, and from 37 to 76 years in females. There were 55 males and 49 females. The sex incidence ratio was male to female 1·1 : 1.

Fig 1.—The age and sex incidence of patients.

*Racial incidence.*—All but 2 patients were either pure negroes or of predominantly negro origin. The remaining 2 were a white female and a Chinese male. This is consistent with the racial distribution of the Jamaican population.

*Duration of symptoms.*—The duration of symptoms was 6 months or less in 80% of cases. In 3 cases the disease was discovered as an incidental finding.

*Presenting symptoms.*—The most frequent presenting symptom was pain, which was present in 87% of cases. The most frequent site was the back, particularly the lumbar and lower thoracic regions. Weakness, malaise, loss of weight, as well as bleeding manifestations and neurological symptoms were other symptoms.

*Physical signs on admission.*—Pallor indicating anaemia (65%), loss of weight (40%), bone tenderness and pathological fractures (25%) were the most common findings on admission. Evidence of infection (20%), neurological complications (15%), and bleeding manifestations (12·5%) were also common. Hepatomegaly was observed in 36% and splenomegaly in 12% of cases on admission.

### *Investigations on Admission*

### *Haematological*

Anaemia was present on admission in 74% of cases and was severe (below 9 g./100 ml.) in 50%. The anaemia was normochromic and normocytic and occasionally microcytic and hypochromic. Leucoerythroblastic anaemia was present in 21% of cases. Macrocytic anaemia was uncommon.

The white cell count did not show any characteristic changes. Plasma cells were observed in the peripheral blood on admission in 14% of cases. Plasma cell leukaemia was encountered in 3 cases, which were all in the advanced stages of the disease. Erythrocyte sedimentation rate in excess of 35 mm. in one hour (Westergren) was observed in 94% of cases, and in 59% it was in excess of 100 mm.

Bone marrow examination showed infiltration by abnormal plasma cells in varying numbers, from 10% to nearly complete replacement.

### *Biochemical*

Hyperproteinaemia in excess of 8 g./100 ml. was observed in 60% of cases, and hyperglobulinaemia in excess of 4 g./100 ml. in 76%. Serum albumin was reduced below 3·5 g./100 ml. in 70% of cases. An abnormal band on electrophoresis was observed in 90·9% of cases. The abnormal band showed a mobility in the gamma region in 47% of cases, in the beta region in 21·3%, between beta and gamma (" M " band) in 18·1%, and in the alpha-2 region in 4·5% of cases. There was a relationship between the presence of an abnormal globulin with a mobility in the beta region, and a more atypical cytology. There was no correlation between the prognosis and cytology, or electrophoretic mobility.

Bence-Jones protein was detected in 50% of cases. It was detected by electrophoresis of concentrated urine in some cases where the classical heat test was negative. There was no correlation between the type of abnormal globulin and the presence of Bence-Jones proteinuria. Cryoglobulinaemia was observed in only 2 cases, but in view of the high normal temperature, it may not have manifested itself in a number of cases. Hypercalcaemia was observed in 39% of cases. There was good correlation between hypercalcaemia and poor prognosis. The serum inorganic phosphorus was usually normal, except in patients in renal failure. The serum alkaline phosphatase was found to be normal in 87·5% of cases, and two thirds of the cases with raised alkaline phosphatase were found to have large pathological fractures. In some of these cases the alkaline phosphatase returned to normal when the fractures had healed. Serum uric acid was estimated in only a few cases, but it was found to be raised in most of them. Raised blood urea was related to Bence-Jones proteinuria, and was associated with poor prognosis.

### *Radiological*

The majority of patients exhibited radiological abnormalities. These manifested themselves most commonly as generalised osteoporosis, but classical osteolytic lesions were also very common. In 10·6% of cases no radiological abnormality was demonstrable, and in one case osteosclerosis, which is very uncommon in multiple myeloma, was present. Pathological fractures were common, and sometimes the disease first manifested itself in this manner. The fractures were most common in the spine and in the ribs, but were also observed in the long bones. The bone lesions were often very extensive.

### *Survival*

The length of survival, either from the onset of symptoms or from the time of diagnosis, was very short. Fig. 2 shows the length of survival after diagnosis. There was no difference in survival between the sexes, and there was no significant difference in survival between the younger and the older age groups. The average survival from the onset of symptoms was 8·4 months in males, and 6·4 months in females. The average survival from the time of diagnosis was 2·4 months in males, and 2·1 months in females.

Fig. 2.—The length of survival after diagnosis.

### *Post Mortem Findings*

Post mortem examinations were performed in 32 cases. The most common cause of death was bronchopneumonia, and infectious complications were encountered in the majority of cases. Bleeding manifestations were not an uncommon cause of death, and in 3 cases death was due to massive pulmonary embolism, a cause of death not recorded in other reported series. Renal failure was not uncommon, often occurring in conjunction with other complications.

Involvement of the skeletal system was observed in all the cases examined. The extent of the involvement varied, and in some cases the typical lesions had to be searched for. Osteoporosis was very common. Splenic enlargement was observed in 35·7% of cases. Microscopic involvement of the spleen was observed in 75% of cases, but tumour deposits were not observed macroscopically. Hepatic enlargement was observed in 64·3% of cases, and in 53% there was microscopical involvement. In 2 cases tumour nodules simulating carcinomatous metastases were observed macroscopically. Histologically the infiltrates were usually portal in distribution. Enlargement of the intra-abdominal and/or the mediastinal lymph nodes was observed in 46·6% of cases. Histologically lymph node infiltration by atypical plasma cells was observed in 57% of cases. Renal involvement was common. A classical picture of " myeloma kidney " was observed in

66% of cases. In 2 cases there were nodular tumour deposits observed macroscopically, and 39% of cases exhibited microscopical involvement. In 32% of cases there was evidence of either chronic or acute pyelonephritis, and in some of these cases there were multiple small cortical abscesses. Amyloid deposition was present in 21% of the kidneys examined. Extra-medullary lesions were observed in many other sites, and their distribution is shown in Fig. 3.

Fig. 3.—The sites of extra-medullary lesions in 28 patients examined post mortem. The stippled column shows the number of cases exhibiting amyloidosis.

Amyloid deposits were observed in 7 cases examined post mortem (21%). In 4 cases there was widespread involvement corresponding both to primary and secondary amyloidosis. Two cases showed the pattern of primary amyloidosis, and in one case there were deposits in the kidneys. Six patients with amyloidosis were aged between 40 and 50 years, only 2 patients complained of bone pain, and 5 patients died within one week of admission. Severe bleeding manifestations were the cause of death in 3 of these patients. There was only slight bone involvement in the patients exhibiting amyloidosis. There was no correlation between amyloidosis and the mobility of the abnormal globulin on electrophoresis.

There was no increased incidence of other primary malignancies in patients with multiple myeloma. One patient had previously been treated for carcinoma of the cervix. One case of multiple myeloma associated with polycythaemia was observed, and 2 cases exhibited myelofibrosis. Familial incidence was observed in one instance, a mother and son being affected. In one case multiple myeloma was diagnosed in the last trimester of pregnancy. The patient had a normal full term delivery, and has survived 3 years after diagnosis. The child is developing normally. One possible case of " pre-myeloma " was encountered.

Solitary plasmocytoma was found to be very uncommon in Jamaica. Two cases of solitary intra-osseous plasmocytoma were encountered, and both showed dissemination of the disease after an interval of some years, thus confirming the close association between solitary plasmocytoma and multiple myeloma. In one of these cases dissemination occurred 9 years after diagnosis. The only patient with solitary extra-osseous plasmocytoma was well with no recurrence of the lesion 2 years after resection of a nasal tumour, and was then lost to follow-up. The prognosis in these cases was much better than in multiple myeloma, but when dissemination occurred the prognosis became similar. The rarity of solitary plasmocytoma in Jamaica can be at least partly explained by the fact that patients present late in the course of the disease, and by that time dissemination may already have taken place.

### DISCUSSION

Multiple myeloma occurs in all parts of the world, and in all races (Steiner, 1954), but its incidence varies, being relatively high in Western Europe and North America, and low in Africa, Asia and South America (Doll *et al.*, 1966).

As the Jamaican population is predominantly negro of West African descent, the comparison of the results of this study with the reports of this disease in parts of Africa populated by negroes and with those referring to the American negro may be of special interest. The life expectancy of the Jamaican negro is similar to that of the African negro, especially above the age of 40 years (Bras, 1967).

In the United States the incidence of multiple myeloma in the negro is similar to that in the white population (Gilliam, 1953; Carson *et al.*, 1955; Kenny and Moloney, 1957; Study Committee, 1964; McMahon, 1966). McMahon and Clark (1956) considered that the incidence was higher in the negro.

The reports from parts of Africa populated by negroes indicate that the incidence of multiple myeloma is low. The disease is very rare in Nigeria (Edington and Maclean, 1965; McFarlane, 1967, personal communication). Payet *et al.* (1963) collected 38 cases from the French speaking West and Central Africa during a 12 year period. In East Africa the disease is also uncommon. Gelfand (1961) has not encountered the disease in Rhodesia, and Davies *et al.* (1965) encountered only 15 cases in the whole of Uganda in 7 years. Eighteen cases have been recorded in Kenya Africans by Linsell (1967) between 1957–63.

The results of the present study, as well as those of the Jamaica Cancer Registry (Bras *et al.*, 1965) indicate that the incidence of multiple myeloma in Jamaica is much higher. But it is by no means as high as has been claimed by McFarlane (1966), who suggested that the incidence of the disease in Jamaica may be among the highest in the world.

The age incidence in the present study was similar to that reported in the recent large series from Europe and North America (Martin, 1961; Study Committee, 1964; Griffiths, 1966; Nordenson, 1966; Carbone *et al.*, 1967). It has been stated by McMahon and Clark (1956) as a result of a study of a small number of cases in Brooklyn, New York, that the disease occurs earlier in the negro than in white population. But the only other study of multiple myeloma in the American negro (Rosenbaum *et al.*, 1958) from Harlem Hospital, New York, did not support these observations. The results of the present study also do not support the findings of McMahon and Clark (1956), in spite of the fact that the majority of the Jamaican population is young.

The sex incidence in the present study, which was practically equal, is in accordance with other recently reported series (Martin, 1961; Griffiths, 1966; Nordenson, 1966; Carbone *et al.*, 1967), and differs from the earlier findings, which showed marked male predominance in the ratio of 2–3 : 1 (Wallgren, 1921; Geschicter and Copeland, 1928; Atkinson, 1937).

The clinical findings in the present study were similar to the findings of other recent investigators (Snapper *et al.*, 1953; Carson *et al.*, 1955; Kenny and Moloney, 1957; Innes and Newall, 1961; Study Committee, 1964; Griffiths, 1966; Nordenson, 1966; Carbone *et al.*, 1967), but many patients were admitted in more advanced stages of the disease.

The anaemia on admission was more severe than in the reports mentioned above, as half the patients had haemoglobin concentration below 9 g./100 ml. As the absence of anaemia on admission was considered to be one of the important prognostic parameters by Carbone *et al.* (1967), the severity of anaemia in the present study correlates well with the poor prognosis.

The biochemical results including serum electrophoresis were similar to those reported from Europe and North America (Adams *et al.*, 1949; Snapper *et al.*, 1953; Osserman, 1959; Innes and Newall, 1961; Martin, 1961; Nordenson, 1966). This is of interest, as Payet *et al.* (1963) from Dakar have stated that the electrophoretic pattern in the African is different, the abnormal band being usually found in the alpha-2 region. The results of the present study also differ from those of McFarlane (1966) in Jamaica, who stated that the abnormal band on electrophoretogram was found to be more common in the beta and alpha-2 regions than in other reported series. As his study was based on a smaller number of cases, this is considered to be at least partly due to selection. Immunoglobulin studies in patients with multiple myeloma in Jamaica (McFarlane *et al.*, unpublished) show similarity to results obtained in the recent studies from Europe and North America (Martin, 1961; Waldenstrom, 1961; Osserman and Takatsuki, 1963; Alper *et al.*, 1966; Carbone *et al.*, 1967).

The radiological findings did not differ markedly from those reported by other investigators (Heiser and Schwartzmann, 1952; Snapper *et al.*, 1953; Carson *et al.*, 1955; Griffiths, 1966). The lesions were similar, but their extent on admission was often more widespread and the incidence of pathological fractures was higher.

The prognosis in the present study was poor as compared with recent reports from Europe and North America (Snapper *et al.*, 1953; Carson *et al.*, 1955; Videbaek and Johansen, 1956; Study Committee, 1964, and Nordenson, 1966), but was similar to that reported from Harlem Hospital, New York (Rosenbaum *et al.*, 1958) and from Dakar, West Africa (Payet *et al.*, 1963). It is not suggested that the disease has a more rapid course in the negro, but these findings only reflect the late presentation of patients, the more advanced stage of the disease, and to some extent lack of the more modern therapeutic facilities. Extra-medullary involvement at post mortem was common in the present study, supporting the observations of recent investigators (Lowenhaupt, 1945; Churg and Gordon, 1950; Hayes *et al.*, 1952; Shapiro and Watson, 1953; Snapper *et al.*, 1953, and Carson *et al.*, 1955), who have emphasised the widespread extra-medullary involvement produced by the disease, which earlier was considered to be very uncommon (Geschicter and Copeland, 1928). These findings contradict the results of Payet *et al.* (1963) based on a small number of cases, stating that extra-medullary involvement is not observed in the negro. The results of the present study support the findings of other investigators (Lichtenstein and Jaffe, 1947; Snapper *et al.*, 1953, Carson *et al.*, 1955; Innes and Newall, 1961; Griffiths, 1966; Rappaport, 1966) concerning the better prognosis of solitary plasmocytoma, and the fact that it is closely related to multiple myeloma.

### SUMMARY

A clinico-pathological study of multiple myeloma in Jamaica was undertaken in order to examine the disease pattern in a predominantly negro population of West African origin. During a 10-year period (1957–66) 101 cases of multiple myeloma and 3 cases of solitary plasmocytoma were encountered. This indicates that multiple myeloma is more common in Jamaica than in parts of Africa populated by negroes. The sex incidence was nearly equal, and there was no evidence that the disease occurs earlier in the negro than in white populations. The haematological, biochemical, and radiological findings were similar to those reported from Europe and North America. The majority of patients presented with advanced disease, and the prognosis was very poor. Post mortem findings showed a high incidence of extra-medullary involvement and amyloidosis.

This study formed part of a thesis submitted to the University of Sheffield for the degree of Doctor of Medicine.

### REFERENCES

Adams, W. S., Alling, E. L. and Lawrence, J. S.—(1949) *Am. J. Med.*, **6**, 141.
Alper, C. A., Rosen, F. S. and Janeway, C. A.—(1966) *New Engl. J. Med.*, **275**, 591.
Atkinson, F. R. B.—(1937) *Med. Press*, **195**, 312, 327.
Bayrd, E. D. and Heck, F. J.—(1947) *J. Am. med. Ass.*, **133**, 147.
Bras, G.—(1967) Proceedings of the 12th Scientific Meeting of the Standing Advisory Committee for Medical Research in the British Caribbean.
Bras, G., Watler, D. C. and Ashmeade-Dyer, A.—(1965) *Br. J. Cancer*, **19**, 681.
Carbone, P. P., Kellerhouse, L. E. and Gehan, E. A.—(1967) *Am. J. Med.*, **42**, 937.
Carson, C. P., Ackerman, L. V. and Maltby, J. D.—(1955) *Am. J. clin. Path.*, **25**, 849.
Churg, J. and Gordon, A.—(1950) *Am. J. clin. Path.*, **20**, 934.
Davies, J. N. P., Knowelden, J. and Wilson, B. A.—(1965) *J. natn. Cancer Inst.*, **35**, 789.
Doll, R., Payne, P. and Waterhouse, J., Editors (1966) ‘ Cancer Incidence in Five Continents ’. Publication of the International Union against Cancer. Berlin (Springer-Verlag).
Edington, G. M. and Maclean, E.—(1965) *Br. J. Cancer*, **19**, 471.
Gelfand, M.—(1961) ‘ Medicine in Tropical Africa ’. Edinburgh (E. & S. Livingstone).
Geschicter, C. F. and Copeland, M. M.—(1928) *Archs Surg., Chicago*, **16**, 807.
Gilliam, A. G.—(1953) *Blood*, **8**, 693.
Griffiths, D. L.—(1966) *J. Bone Jt Surg.*, **48**-B, 703.
Hayes, D. W., Bennett, W. A. and Heck, F. J.—(1952) *A.M.A. Archs Path.*, **53**, 262.
Heiser, S. and Schwartzmann, J. J.—(1952) *Radiology*, **58**, 179.
Innes, J. and Newall, J.—(1961) *Lancet*, i, 239.
Kenny, J. J. and Moloney, W. C.—(1957) *Ann. intern. Med.*, **46**, 1079.
Lichtenstein, L. and Jaffe, H. L.—(1947) *Archs Path.*, **44**, 207.
Linsell, C. A.—(1967) *Br. J. Cancer*, **21**, 465.
Lowenhaupt, E.—(1945) *Am. J. Path.*, **21**, 171.
McFarlane, H.—(1966) *J. clin. Path.*, **19**, 268.
McMahon, B.—(1966) *Cancer Res.*, **26**, 1189.

McMahon, B. and Clark, D. W.—(1956) *J. chron. Dis.*, **4**, 508.
Martin, N. H.—(1961) *Lancet*, i, 237.
Nordenson, N. G.—(1966) *Acta med. scand.*, Suppl., **445**, 178.
Osserman, E. F.—(1959) *New Engl. J. Med.*, **261**, 952, 1006.
Osserman, E. F. and Takatsuki, K.—(1963) *Medicine, Baltimore*, **42**, 357.
Payet, M., Sankale, M., Pene, P. and Diop, B.—(1963) *Bull. Soc. méd. Afr. noire Lang. fr.*, **8**, 338.
Rappaport, H.—(1966) 'Tumours of the Hematopoietic System'. 'Atlas of Tumor Pathology'. Section 3. Fascicle 8. Washington, D.C. (Armed Forces Institute of Pathology).
Rosenbaum, L., Marquez, C. and Galarraga, E. C.—(1958) *J. natn. med. Ass.*, **50**, 344.
Shapiro, H. D. and Watson, R. J.—(1953) *Blood*, **8**, 755.
Snapper, I., Turner, L. B. and Moscovitz, H. L. (1953). 'Multiple Myeloma'. New York (Grune and Stratton).
Steiner, P.—(1954) 'Cancer: Race and Geography'. Baltimore (The Williams and Wilkins Co.).
Study Committee of the Midwest Cooperative Chemotherapy Group (Best, W. R. *et al.*)—(1964) *J. Am. med. Ass.*, **188**, 741.
Videbaek, A. and Johansen, H.—(1956) *Dan. med. Bull.*, **3**, 174.
Waldenstrom, J.—(1960) *Proc. R. Soc. Med.*, **53**, 789.—(1961) *Acta med. scand.*, **170**, Suppl. 376.
Wallgren, A. B.—(1921) *Virchows Arch. path. Anat. Physiol.*, **232**, 381.